# Large-scale identification of *Gossypium hirsutum* genes associated with *Verticillium dahliae* by comparative transcriptomic and reverse genetics analysis

**DOI:** 10.1371/journal.pone.0181609

**Published:** 2017-08-02

**Authors:** Wenwei Zhang, Huachong Zhang, Kai Liu, Guiliang Jian, Fangjun Qi, Ning Si

**Affiliations:** State Key Laboratory for Biology of Plant Diseases and Insect Pests, Institute of Plant Protection, Chinese Academy of Agricultural Sciences, Beijing, P. R. China; USDA-ARS Southern Regional Research Center, UNITED STATES

## Abstract

Verticillium wilt is a devastating disease of cotton, which is caused by the soil-borne fungus *Verticillium dahliae* (*V*. *dahliae*). Although previous studies have identified some genes or biological processes involved in the interaction between cotton and *V*. *dahliae*, its underlying molecular mechanism remains unclear, especially in *G*. *hirsutum*. In the present study, we obtained an overview of transcriptome characteristics of resistant upland cotton (*G*. *hirsutum*) after *V*. *dahliae* infection at 24 h post-inoculation (hpi) via a high-throughput RNA-sequencing technique. A total of 4,794 differentially expressed genes (DEGs) were identified, including 820 up-regulated genes and 3,974 down-regulated genes. The enrichment analysis showed that several important processes were induced upon *V*. *dahliae* infection, such as plant hormone signal transduction, plant-pathogen interaction, phenylpropanoid-related and ubiquitin-mediated signals. Moreover, we investigated some key regulatory gene families involved in the defense response, such as receptor-like protein kinases (RLKs), WRKY transcription factors and cytochrome P450 (CYPs), via virus-induced gene silencing (VIGS). GhSKIP35, a partner of SKP1 protein, was involved in ubiquitin-mediated signal. Over-expression of GhSKIP35 in *Arabidopsis* improved its tolerance to Verticillium wilt in transgenic plants. Collectively, global transcriptome analysis and functional gene characterization provided significant insights into the molecular mechanisms of *G*. *hirsutum*-*V*. *dahliae* interaction and offered a number of candidate genes as potential sources for breeding wilt-tolerance in cotton.

## Introduction

Cotton (*Gossypium* spp.) is a widely cultivated plant and has important economic value because of its fiber and oil. In many cotton diseases, Verticillium wilt is one of the most devastating diseases of cotton worldwide. In China, Verticillium wilt affects more than 70% of cotton planting areas, leading to huge economic loss every year. Verticillium wilt is caused by the soil-borne fungus *Verticillium dahliae* Kleb. It is difficult to control this pathogen, due to its long-term survival as microsclerotia in the soil and broad host range. The primary strategies mainly include breeding and cultivation of resistant varieties. *G*. *hirsutum* is known as upland cotton, and it produces more than 95% of the annual cotton crop worldwide. However, the underlying genetic and molecular mechanisms of cotton resistance to Verticillium infection remain poorly explored.

Previous studies have mainly focused on the molecular characterization of the defense responses of *G*. *barbadense* upon *V*. *dahliae* infection. The phenylpropanoid pathway plays a key role during the plant defense response to *V*. *dahliae* [1], which has been also confirmed in tomato [2] and pepper [3]. Xu et al. [1] performed RNA-sequencing in island cotton variety (*G*. *barbadense*) and identified the central role of lignin metabolism in cotton resistance to *V*. *dahliae*. The lignin level is also detected by histochemical analysis, showing that lignin levels are higher in resistant plants compared with susceptible plants. Ethylene (ET) has been shown to have dual roles in resistance to Verticillium wilt. ET biosynthesis and signaling components are activated in response to *V*. *dahliae* infection, which can trigger the systemic resistance in plants [4]. However, former study has shown that released ET makes cotton susceptible to Verticillium wilt, resulting in wilting leaves [5]. It has been identified that several pathogenesis-related (PR) proteins, such as Bet v1 family and major latex protein (MLP), play important roles in defense reaction against Verticillium wilt [4, 6, 7, 8]. These findings indicated that cotton defense response to *V*. *dahliae* infection is sophisticated, which might involve different defense pathways.

Several previous studies have characterized transcriptomic changes in the cotton defense response to *V*. *dahliae* infection [1, 9, 10]. Our group has identified 99 differentially expressed genes (DEGs) in resistant *G*. *hirsutum* varieties compared with susceptible varieties by suppression subtractive hybridization (SSH) [7]. But, these data are not enough to elucidate the defense mechanism of cotton against *V*. *dahliae*. Moreover, a comprehensive understanding of molecular mechanisms of cotton defense response to *V*. *dahliae* has not been established due to complexity of cotton genome. Genome-wide transcriptome analysis is a potentially valuable strategy to elucidate the underlying molecular mechanisms of the physiological processes, and it is substantially efficient to identify genes of interest. Moreover, virus-induced gene silencing (VIGS) offers an efficient approach for large-scale functional analysis of individual genes [11], which will elaborate our understanding of cotton defense response to Verticillium wilt.

In the present study, we aimed to deepen our understanding of upland cotton defense mechanisms against *V*. *dahliae*. A comprehensive transcriptome analysis of upland cotton under *V*. *dahliae* attack was performed via high-throughput RNA-sequencing technology. Furthermore, in combination with VIGS technique, we further characterized DEGs functions against *V*. *dahliae* in upland cotton. Our present data provided valuable information on the molecular basis of cotton defense against *V*. *dahliae* and also provided some important candidate genes against *V*. *dahliae*.

## Materials and methods

### Plant materials and pathogen inoculation

Two varieties of upland cotton (*G*. *hirsutum*), including Zhongzhimian KV3 (Verticillium wilt-resistant upland cotton) and 86–1 (Verticillium wilt-susceptible upland cotton) were used in the present study. Seed germination and seedling growth were carried out at day/night temperatures of 26/20°C with 1/3 MS medium [12] in sterile culture pots under long-day condition (16-h photoperiod) until the second true leaves appeared. Cotton seedlings were inoculated using root dipping method [7]. Inoculation was performed using the highly toxic and defoliant strain of *V*. *dahliae*, V991. Conidial production was prepared based on a previously described method [7], and the concentration of the conidial suspension was adjusted to 1.0×10^7^ conidia/mL. The root tissues were harvested at 24 h post-inoculation (hpi). While additional seedlings were mock-inoculated appropriately with sterile distilled water as non-inoculated controls. More than 10 plants per treatment were collected and then immediately frozen in liquid nitrogen.

### Library construction and Illumina sequencing

Total RNA was extracted using a modified cetyltrimethylammonium bromide (CTAB) method. The integrity of RNA was assessed using denaturing agarose gel electrophoresis and spectrophotometrically examined according to the ratio of A260/A280 (Perkin-Elmer, USA). The RNA samples, including samples of Zhongzhimian KV3 infected with *V*. *dahliae* strain V991 at 24 h and mock-inoculated samples as non-inoculated controls, were sent to Beijing Genomic Company (BGI, Shenzhen, China). The cDNA library construction and Illumina-base paired-end deep sequencing were performed following the manufacturer’s instructions (Illumina). Two biological replicates were used for all RNA-sequencing experiments. Raw reads produced by Illumina HiSeq 2000 were filtered into clean reads by removing reads containing adapter, unknown bases or low quality reads. After screening, clean reads were aligned to the reference sequences with SOAP aligner/SOAP2 [13]. Distribution of reads was determined according to data alignment, and coverage analysis was then performed.

### Gene expression and data analysis

The gene expression level was calculated and normalized by using RPKM (reads per kilobase transcriptome per million mapped reads) method [14]. DEGs of replicates between inoculated and mock-inoculated groups were performed using the DESeq R package (1.10.1) based on the negative binomial distribution. The P-values were adjusted using the Benjamini and Yekutieli’s method [15] to control the false discovery rate (FDR). Genes with FDR≤0.01 and the absolute value of Log_2_Ratio≥2 were used as the threshold to identify the significant DEGs [16](Audic et al., 1997). Moreover, functions of DEGs were classified by gene ontology (GO) enrichment analysis and pathway assignments in KEGG (Kyoto Encyclopedia of Genes and Genomes) database [17,18].

### qRT-PCR analysis

Total RNA was isolated as previously described. Purified RNA was treated with DNase I (TaKaRa, Japan), then reversely transcribed into cDNA using M-MLV Reverse Transcriptase (Promega, USA) and quantified with spectrophotometry (Perkin-Elmer, USA). Quantitative amplification was performed in triplicate using SuperReal PreMix Plus (Tiangen, China) on an ABI 7500 Real Time PCR System. Briefly, after an initial denaturation step at 95°C for 15 min, the amplification was carried out with 40 cycles at a denaturating temperature of 95°C for 10 sec, and an annealing temperature of 60–62°C for 32 sec. Cotton ubiquitin gene (GenBank EU604080) or *Arabidopsis* actin gene (GenBank AY064043.1) was used as an endogenous reference for data normalization, and S1 and S3 Tables list the primer sequences used in the present study. The relative expression of the target genes at the mRNA level was determined using the 2^−ΔΔCt^ method [19]. Relative gene expression was analyzed by one-way ANOVA using the SAS 8.1. The confidence level of all analyses was set at 95%, and P<0.05 was considered statistically significant.

### Gene function analysis by VIGS

pCLCrVA and pCLCrVB, the cotton leaf crumple virus (CLCrV)-based vectors, were used for VIGS [11]. RT-PCR was employed to amplify the candidate genes selected from DEGs (primers were listed in S2 Table), and then the amplicons were inserted into pCLCrVA vector. Moreover, pCLCrVA, pCLCrVB and derivatives of pCLCrVA were respectively transferred into *Agrobacterium tumefaciens* EHA105 via electroporation. The solution of *Agrobacterium* harboring pCLCrVA or one of its derivatives was mixed with an equal volume of *Agrobacterium* harboring pCLCrVB. Then, mixture solution was infiltrated into two fully expanded cotyledons of 10-day-old upland cotton seedlings (Zhongzhimian KV3 and 86–1) as previously described [20]. The CLA1 (endogenous cloroplastos alterados 1) gene was used as the control in VIGS, which encoded 1-deoxyxylulose 5-phosphate synthase, the first enzyme of the 2-C-methyl-D-erythritol-4-phosphate pathway involved in chloroplast development [21]. Each experiment was performed at least in triplicate with more than 20 plants for each construct per repeat. The corresponding primers were used to determine the expression levels of target genes in the leaf tissues of cotton seedling 20 days after VIGS treatment (S3 Table).

### *Arabidopsis* transformation

The coding sequence of GhSKIP35 was cloned into the plant expression vector pPZP111 [22]. The resulting plasmid pPZP111-GhSKIP35 was introduced into *A*. *tumefaciens* strain EHA105. *Agrobacterium*-mediated flower dipping transformation method was used to generate transgenic *Arabidopsis* plants [23], which were then selected on kanamycin and confirmed by qRT-PCR. Total RNA of wild-type *Arabidopsis* plants and GhSKIP35 transgenic plants were extracted by TRIzol method. Purified RNA was then reversely transcribed into cDNA. *Arabidopsi*s actin gene (GenBank AY064043) was selected as an endogenous reference gene (primer sequences were listed in S3 Table). qRT-PCR was performed as above mentioned.

### Assessment of disease development and statistical analysis

When GhCLA1-silenced plants exhibited the photobleaching phenotype at 20 days after *Agrobacterium* infiltration, the other target gene silenced plants and negative control plants were subjected to *V*. *dahliae* infection by root dipping in conidial suspension (10^7^ conidia/mL) for 30 min. In order to assess the disease development, the plants were then replanted in fresh soil. According to the percentage of affected plant tissues, such as chlorosis, necrosis, wilt and defoliation, a Verticillium wilt disease index (DI) scaling from 0 to 4 was introduced to evaluate the disease severity [24]. The DI of Verticillium wilt was determined using the following formula:

$$\text{DI}=\frac{\sum \left(\text{n}\times \text{number of plants at level n}\right)}{4\times \text{number of total plants}}\times 100$$

Data from three separate experiments were analyzed by ANOVA using the SAS 8.1. The confidence level of all analyses was set at 95%, and P < 0.05 was considered as statistically significant.

## Results

### Variety Zhongzhimian KV3 shows high resistance to *V*. *dahliae* inoculation

The highly toxic and defoliant pathogenic *V*. *dahliae* strain (V991) was used to inoculate the upland cotton variety Zhongzhimian KV3 (resistant) and 86–1 (susceptible). The disease index (DI) of Zhongzhimian KV3 and 86–1 are 5.303 ± 0.35 and 47.27±2.79 _in the artificial disease nursery, respectively [8, 24]. Our data showed significant difference between susceptible and resistant plants in terms of disease symptoms and growth status at 10 days post inoculation (dpi) in the green house. In addition, we reproducibly observed such a process of symptom development after the *V*. *dahliae* infection.

### Transcriptome characterization of Zhongzhimian KV3 infected with *V*. *dahliae* by high-throughput RNA-sequencing

To globally identify resistance genes of upland cotton against *V*. *dahliae*, we used the highly resistant variety Zhongzhimian KV3 to perform comparative transcriptomics analyses. As the first barrier against *V*. *dahliae* invasion, the root tissues inoculated with *V*. *dahliae* strain V991 for 24 h and the mock-inoculated root tissues were used to construct cDNA libraries, which were sequenced by Illumina platform. After filtration of raw reads and quality control, about 52 millions of clean reads were generated from each sample, which was sufficient for quantitative analysis of gene expression. To reveal the molecular events behind the gene expression, it is an important step to match the clean reads to genes in order to annotate sequences [25]. In the present study, all clean reads were aligned to the reference genome (http://cgp.genomics.org.cn/page/species/ContactServlet). S4 Table shows the summarized information of the reads.

A total of 31,319 DEGs were detected during the cotton defense response to *V*. *dahliae* using RPKM-based method. Moreover, 4,794 DEGs were obtained using FDR≤0.01 and the absolute value of Log_2_Ratio≥2 as the threshold. Among them, 820 up-regulated genes and 3,974 down-regulated genes were screened, which might be involved in the *V*. *dahliae*-*G*. *hirsutum* interaction. According to the DEGs expression pattern upon *V*. *dahliae* infection, down-regulated model contained the maximum DEGs, showing that negative regulation might play important roles against Verticillium wilt in cotton.

### GO clustering and pathway enrichment analysis of DEGs

**Fig 1:**
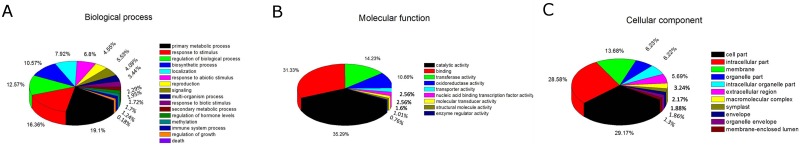
Functional classifications of differentially expressed genes (DEGs) were determined by GO terms. The GO categories are presented as follows: A, biological process, B, molecular function, C, cellular component. Pie charts represent the functional distribution, which is expressed as a percentage of the amount of genes.

To better recognize the main biological functions of DEGs, GO was used to comprehensively describe properties of genes, which were divided into various categories, including cellular component (3,025 DEGs), molecular function (2,770 DEGs) and biological process (3,026 DEGs) (Fig 1). ‘Primary metabolic process’ and ‘response to stimulus’ were the most highly represented groups in the biological process category, which might be a rapid response of cotton to pathogen. Genes involved in other important biological processes, such as biosynthetic process (10.57%), response to abiotic stimulus (6.8%), signaling (5.53%) and response to biotic stimulus (3.44%), were also identified. In the molecular function category, the two most abundant sub-categories were catalytic activity (35.29%) and binding (31.33%). In the cellular component category, a large number of unigenes were categorized into cell part (29.17%), intracellular part (28.58%) and membrane (13.68%).

In addition, 2,925 DEGs were assigned to 127 KEGG pathways. Table 1 lists the pathways with over 70 representatives. Among these pathways, ‘metabolic pathways’ (744 DEGs, 26.46%) and ‘biosynthesis of metabolites’ (437 DEGs, 14.94%) possessed the most members in all pathways. Moreover, ‘plant-pathogen interaction’ (295 DEGs, 10.09%) and ‘plant hormone signal’ (293 DEGs, 10.02%) pathways played important roles in response to Verticillium wilt.

**Table 1:** KEGG (Kyoto Encyclopedia of Genes and Genomes) pathway analysis of differentially expressed genes (DEGs).

No.	Pathway	DEG numbers (percent)	Pathway ID
1	Metabolic pathways	774 (26.46%)	ko01100
2	Biosynthesis of secondary metabolites	437 (14.94%)	ko01110
3	Plant-pathogen interaction	295 (10.09%)	ko04626
4	Plant hormone signal transduction	293 (10.02%)	ko04075
5	Starch and sucrose metabolism	95 (3.25%)	ko00500
6	Glycerophospholipid metabolism	91 (3.11%)	ko00564
7	Phenylpropanoid biosynthesis	84 (2.87%)	Ko00940
8	Endocytosis	79 (2.70%)	ko04144
9	Stilbenoid, diarylheptanoid and gingerol biosynthesis	74 (2.53%)	Ko00945
10	Ether lipid metabolism	71 (2.43%)	ko00565
11	Flavonoid biosynthesis	70 (2.39%)	Ko00941
12	Pentose and glucuronate interconversions	70 (2.39%)	ko00040

### Validation of RNASeq by qRT-PCR

**Fig 2:**
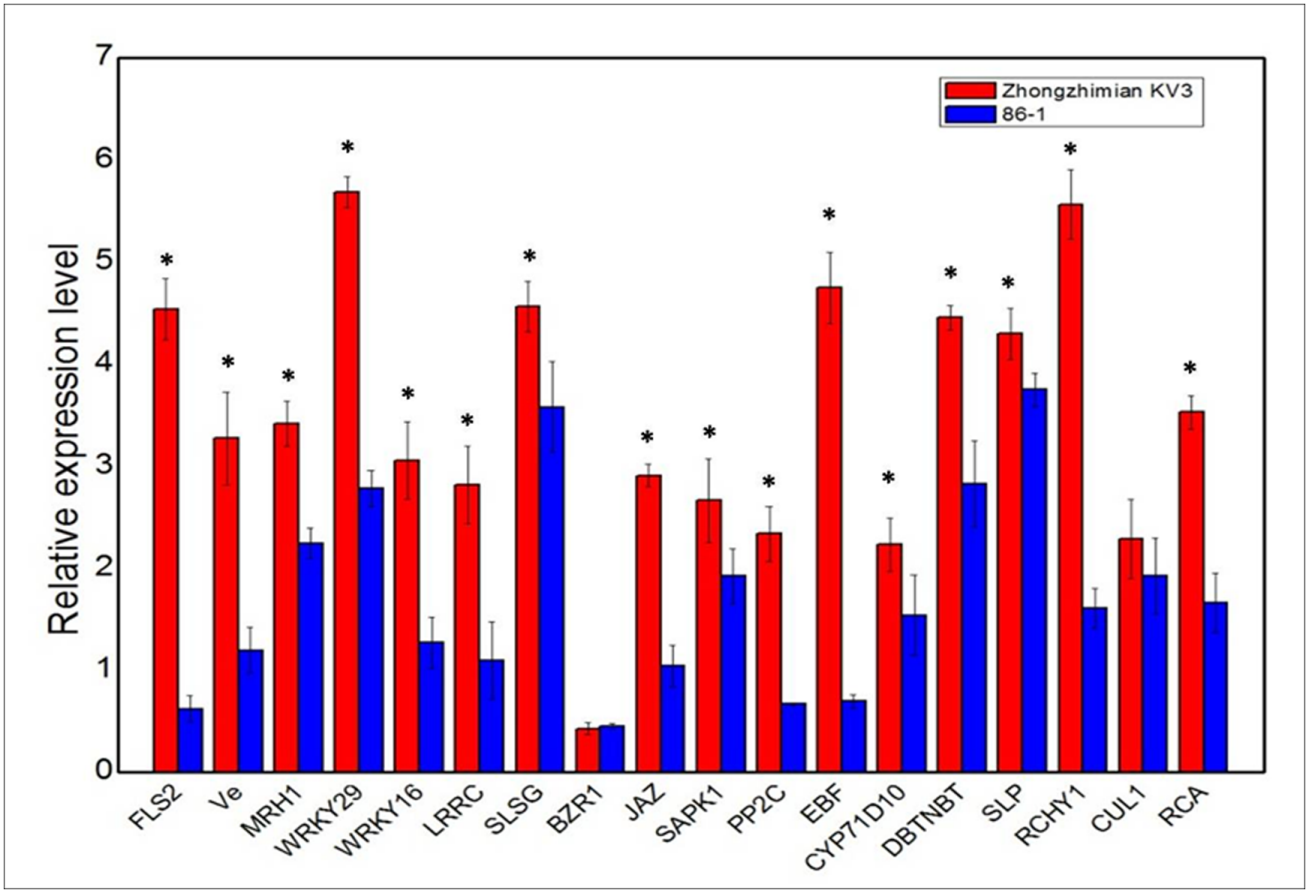
The quantitative real-time PCR analysis of 18 differentially expressed genes (DEGs) between resistant upland variety Zhongzhimian KV3 and susceptible upland variety 86–1 at 24 h post inpculation (hpi) with *V*. *dahliae*. The relative gene expression levels of cotton genes induced by *V*. *dahliae* were normalized against Ct values for cotton ubiquitin gene, which were calculated by the 2^−ΔΔCt^ method (Livak & Schmittgen, 2001). The data are mean values and standard deviation (bar) of three independent qRT-PCR experiments. Asterisk represents the data point of Zhongzhimian KV3 that was statistically different from the data point of 86–1 (p<0.05) analysed by one-way ANOVA, using the SAS 8.1. The vertical bars indicate standard Deviation.

To further validate the reliability of the Illumina sequencing analysis, 18 DEGs were randomly selected for further analysis using qRT-PCR. Their expression trends (Table 2) were almost consistent with the transcriptome sequencing analysis. Among the 18 DEGs, seven DEGs were involved in plant-pathogen interaction pathway and expressed at higher levels than those in non-inoculated plants. Five DEGs were predicted to be involved in plant hormone signal transduction pathway, that jasmonate ZIM domain-containing protein, serine/threonine-protein kinase SAPK1, and protein phosphatase 2 were up-regulated upon *V*. *dahliae* infection, while Brassinazole-resistant 1 protein and EIN3-binding F-box protein were down-regulated. Other DEGs, cytochrome P450 71D10 and 3'-N-debenzoyl-2'-deoxytaxol N-benzoyltransferase related to phenylpropanoid biosynthesis pathway, spotted leaf protein, RING finger, CHY zinc finger domain-containing protein 1 and cullin 1 involved in ubiquitin-mediated proteolysis, ribulose bisphosphate carboxylase/oxygenaseactivase 1 involved in proteasome pathway, were all up-regulated in resistant variety Zhongzhimian KV3 compared with susceptible variety 86–1 upon *V*. *dahliae* infection (Fig 2).

**Table 2:** Quantitative real-time PCR analysis of 18 candidate genes.

Candidate gene	Cotton gene ID	Fold change of qPCR	Nr description	Involved pathway
GhFLS2	Cotton_D_gene_10028922	4.54	LRR receptor-like serine/threonine-protein kinase FLS2	Plant-pathogen interaction
GhVe	Cotton_D_gene_10010079	3.27	GbVe	Plant-pathogen interaction
GhMRH1	Cotton_D_gene_10001059	3.42	probable LRR receptor-like serine/threonine-protein kinase MRH1-like	Plant-pathogen interaction
GhWRKY29	Cotton_D_gene_10024748	5.69	WRKY transcription factor 29	Plant-pathogen interaction
GhWRKY16	Cotton_D_gene_10036575	3.06	WRKY transcription factor 16	Plant-pathogen interaction
GhLRRC	Cotton_D_gene_10022349	2.82	leucine-rich repeat-containing protein	Plant-pathogen interaction
GhSLSG	Cotton_D_gene_10020602	4.57	S-locus-specific glycoprotein S6 precursor	Plant-pathogen interaction
GhBZR1	Cotton_D_gene_10036644	-2.33	Brassinazole-resistant 1 protein	Plant hormone signal transduction
GhJAZ	Cotton_D_gene_10010230	2.91	jasmonate ZIM domain-containing protein	Plant hormone signal transduction
GhSAPK1	Cotton_D_gene_10008368	2.67	Serine/threonine-protein kinase SAPK1	Plant hormone signal transduction
GhPP2C	Cotton_D_gene_10011899	2.34	protein phosphatase 2c	Plant hormone signal transduction
GhEBF	Cotton_D_gene_10016134	-2.86	EIN3-binding F-box protein	Plant hormone signal transduction
GhCYP71D10	Cotton_D_gene_10006923	2.23	P450 71D10	Phenylpropanoid biosynthesis
GhDBTNBT	Cotton_D_gene_10002169	4.46	3'-N-debenzoyl-2'-deoxytaxol N-benzoyltransferase	Phenylpropanoid biosynthesis
GhSLP	Cotton_D_gene_10020522	4.30	Spotted leaf protein	Ubiquitin mediated proteolysis
GhRCHY1	Cotton_D_gene_10005965	5.57	RING finger and CHY zinc finger domain-containing protein 1	Ubiquitin mediated proteolysis
GhCUL1	Cotton_D_gene_10009145	2.29	cullin 1	Ubiquitin mediated proteolysis
GhRCA	Cotton_D_gene_10026030	3.53	Ribulose bisphosphate carboxylase/oxygenase activase 1	Proteasome

### DEGs involved in plant-pathogen interaction pathway are significantly enriched

In the present study, 295 DEGs were found in ‘plant-pathogen interaction’ pathway (Ko0426), including 71 up-regulated genes and 224 down-regulated genes. Among them, genes encoding RLKs made up the largest group, which were up-regulated upon *V*. *dahliae* infection, including leucine-rich repeat receptor-like serine/threonine-protein kinases (At3g14840, At3g47570), receptor-like protein 12, GbVe1, Verticillium wilt resistance-like protein, receptor protein kinase, leucine-rich repeat-containing protein, FLS2, G-type lectin S-receptor-like serine/threonine-protein kinase SD2-5, serine/threonine-protein kinase PBS1, two cysteine-rich receptor-like protein kinases, S-locus-specific glycoprotein S6 precursor, lectin receptor-like kinase and so on. Another important group was WRKY gene family. Several WRKY factors (such as WRKY1, 2, 6, 12, 13, 16, 22, 27, 29, 57 and 74) have been identified, which might be crucial regulators of the defense transcriptome and disease resistance [26]. It has been reported that calmodulins (CaM) can bind to several TGA and WRKY transcription factors that play a role in the activation of stress or defense pathway [27]. Pathogen-associated molecular patterns (PAMPs) can activate Ca^2+^ signals and Ca^2+^-dependent gene expression during plant immune response [27]. In this study, four sub-families of Ca^2+^ sensors including calmodulins (CaM), CaM-like proteins (CMLs), calcium dependent protein kinases (CDPKs) and calcineurin B-like proteins (CBLs) were all identified. The expressions of CML10, CML27, CML38, CBL and EF-hand CBL were up-regulated, while CML22, CML41 and reticulocalbin-2 precursor were down-regulated, indicating that Ca^2+^ signals were tightly related to defense responses upon *V*. *dahliae* invasion in cotton roots. Furthermore, mitogen-actived protein kinase (MAPK) cascades have been shown to be involved in plant signal transduction in response to pathogens [28], and among the DEGs, MAPKK6 (mitogen-activated protein kinase kinase 6) and MAPKKK (mitogen-activated protein kinase kinase kinase) were up-regulated upon pathogen attack.

### WRKYs and RLKs are required for resistance against *V*. *dahliae* in cotton

**Fig 3:**
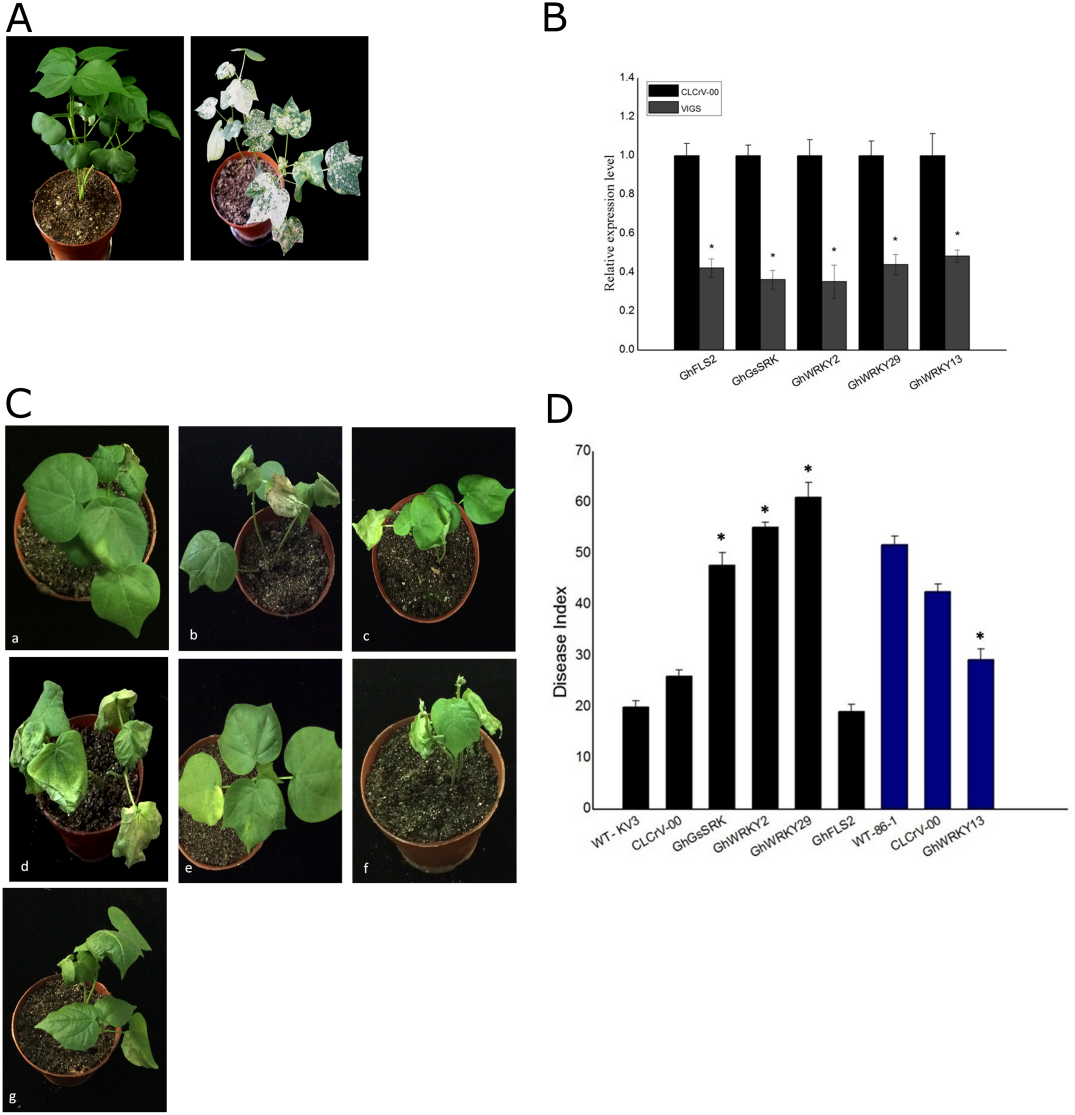
The RLKs and WRKYs are required for resistance against *V*. *dahliae* in cotton via VIGS analysis. A: Silencing of CLA1 gene in *G*. *hirsutum* variety Zhongzhimian KV3. Left, plant infiltrated with CLCrV-based empty vector (CLCrV-00); right, plant infiltrated with CLCrV-CLA1. B: Relative expression levels of candidate gene in the silenced and non-silenced cotton plants at 20 dpi were determined through qRT-PCR. Asterisk represents the data point that was statistically different from the non-silenced (p<0.05) analyzed by one-way ANOVA, using the SAS 8.1. The vertical bars indicate standard Deviation. C: a, Negative control-silenced upland cotton (Zhongzhimian KV3, CLCrV-00); b, GhGsSRK-silenced Zhongzhimian KV3 plant; c, GhWRKY2-silenced Zhongzhimian KV3 plant; d, GhWRKY29-silenced Zhongzhimian KV3 plant; e, GhFLS2-silenced Zhongzhimian KV3 plant; f, Negative control-silenced upland cotton (86–1, CLCrV-00); g, GhWRKY13-silenced 86-1plant. D: The disease index (DI) after silencing different genes. The results are presented as mean ±standard deviation (SD) from three replicates with at least 20 plants per replicate. Black represents candidate gene silenced in Zhongzhimian KV3, and blue represents candidate gene silenced in 86–1. Asterisk represents the data point that was statistically different from wild-type and CLCrV-00 plants (p<0.05) analyzed by one-way ANOVA, using the SAS 8.1. The vertical bars indicate standard Deviation.

Since VIGS technique has been successfully used to describe gene function in cotton, we employed this approach to further assess the roles of three WRKY genes (WRKY2, WRKY29 and WRKY13) and two RLK genes (LRR receptor-like serine/threonine-protein kinase FLS2, GhFLS2 and G-type lectin S-receptor-like serine/threonine-protein kinase GhGsSRK). First, the cotton CLA1 gene was used as a control to monitor the efficiency of VIGS in cotton. By 20 dpi with CLA1 gene silenced plants, the newly emerging leaves showed extensive chlorosis phenotype, while non-silenced plants (plants infected with CLCrV vector without CLA1 gene) exhibited wild-type phenotype (Fig 3A). These findings suggested that the CLCrV vector could effectively silence the endogenous gene in cotton without obvious effect on plant growth. Next, we respectively cloned five candidate gene fragments into pCLCrVA and infiltrated into two fully expanded cotyledons of 10-day-old cotton seedlings together with pCLCrVB at a ratio of 1:1 by *A*. *tumefaciens*, while pCLCrVA empty vector was used as a negative control. At least 20 plants were infiltrated for each construct per repeat. When plants infiltrated with GhCLA1 displayed the photobleaching phenotype at 20 dpi, the top leaves were harvested to determine the expressions of corresponding genes. Compared with the control, the expressions of all five genes were decreased approximately more than 51.61% (Fig 3B), and then the silenced and negative control plants were subjected to *V*. *dahliae* infection. GhFLS2, GhGsSRK, GhWRKY2 and GhWRKY29 were up-regulated upon *V*. *dahliae* infection. We respectively silenced these genes in resistant variety Zhongzhimian KV3. GhGsSRK-, GhWRKY2- and GhWRKY29-silenced plants exhibited more severe symptoms compared with the vector control plants (Fig 3C). The average DI of GhGsSRK-, GhWRKY2- and GhWRKY29-silenced plants was 47.74±2.42, 55.19±0.92 and 61.07±2.83, respectively, while that of control (CLCrV-00) and wild-type plants Zhongzhimian KV3 was 26.04±1.17 and 19.94±1.25, respectively (Fig 3D). However, GhFLS2-silenced plants did not compromise cotton resistance to *V*. *dahliae* (Fig 3C). GhWRKY13 was down-regulated upon *V*. *dahliae* infection. We silenced GhWRKY13 gene in susceptible variety 86–1. Data showed that the GhWRKY13 silencing significantly improved the resistance of susceptible variety to *V*. *dahliae* compared with the control plants (Fig 3C). The average DI of GhWRKY13-silenced plants was 29.24±2.13, while that of control (CLCrV-00) and wild-type plants 86–1 was 51.77±1.66 and 42.57±1.43, respectively (Fig 3D). Taken together, we identified GhGsSRK, GhWRKY2, GhWRKY29 and GhWRKY13 as important components in upland cotton resistance to *V*. *dahliae* infection using VIGS assays.

### Phytohormone signaling-related genes play important roles against *V*. *dahliae* infection

Plant hormones are signal molecules regulating developmental processes, and they modulate signaling networks involved in plant responses to a wide range of biotic and abiotic stresses [29]. In this study, GO and KEGG enrichment analyses of DEGs both highlighted that hormonally regulated genes were particularly affected by the pathogen treatment. In cotton, gene encoding ET signal transduction kinase has different transcriptional expression patterns in the ET signaling pathway upon *V*. *dahliae* infection. DEGs such as ERF domain-containing transcription factor (ERF), ET response element-binding protein (EREBP), ET receptor (ETR) and EIN3-binding F-box protein 1 (EBF1) were up-regulated, while serine/threonine-protein kinase CTR1 (CTR1), ET-insensitive protein 2 (EIN2) and ET-insensitive 3 protein (EIN3) were down-regulated upon *V*. *dahliae* infection. As an endogeneous signal molecule, salicylic acid (SA) can affect plant defense against biotrophic and hemi-biotrophic pathogens and establish the systemic acquired resistance (SAR) [30]. SA-signaling genes, transcription factor TGA (TGA7), pathogenesis-related protein 1 (PR1) and two genes encoding non-expressor of PR protein 1 (NPR1) were identified as suppressed components based on RNA-sequencing. DEGs encoding TIFY5A, TIFY9A and Jasmonate Zim-domain10 (JAZ10) which involved in JA signal were activated in cotton upon *V*. *dahliae*. Moreover, JAZs are proposed to exert their effects on gene expression through interaction with MYC2, one member of the plant basic helix–loop–helix (bHLH) transcription factor family [31]. Four basic bHLH transcription factors, bHLH18, bHLH25, bHLH30 and bHLH84, were activated. Except for above genes involved in defense-related phytohormones, other brassinosteroid- (BR-) and cytokinin-associated genes also responded to pathogen infection. After *V*. *dahliae* infection, BR-resistant protein 1 (BZR1) and BR-resistant protein 2 (BRI2) were repressed, which might play a role in negatively regulating BR biosynthesis. The cytokinin-related gene, glucanendo-1,3-beta-glucosidase 13 gene (E13L13), was up-regulated upon pathogen attack, which might trigger plant defense by hydrolyzing the pathogen fungal cell wall [32].

### Phenylpropanoid pathway-related genes

**Fig 4:**
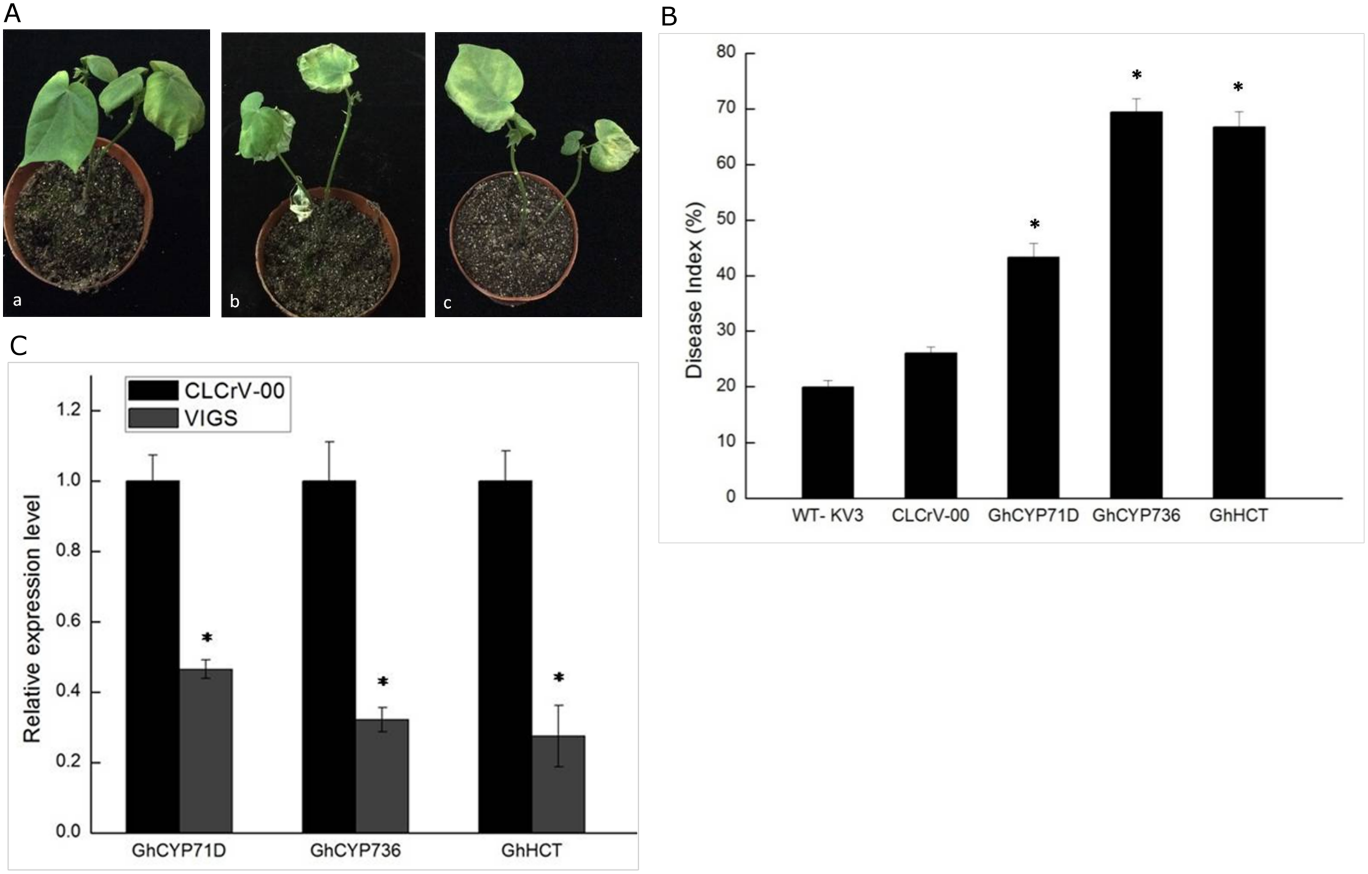
Silencing of phenylpropanoid pathway-related genes in Zhongzhimian KV3 infected with *V*. *dahliae* strain V991. 4A, a, GhCYP71D-silenced Zhongzhimian KV3 plant; b, GhCYP736-silenced Zhongzhimian KV3 plant; c, GhHCT-silenced Zhongzhimian KV3 plant. 4B, The disease index (DI) after silencing different genes. Asterisk represents the data point that was statistically different from wild-type and CLCrV-00 Zhongzhimian KV3 plants (p<0.05) analyzed by one-way ANOVA, using the SAS 8.1. The vertical bars indicate standard Deviation. 4C, Relative expression levels of candidate genes in the silenced and non-silenced cotton plants at 20 dpi were determined through qRT-PCR. Asterisk represents the data point that was statistically different from the non-silenced (p<0.05) analyzed by one-way ANOVA, using the SAS 8.1. The vertical bars indicate standard Deviation.

In cotton, the phenylpropanoid pathway plays an important role in response to *V*. *dahliae*. Cytochrome P450 monooxygenases, including a number of cytochrome P450 (CYP) species, play an important role in the biosynthesis of phenylpropanoids [33]. CYP71D and CYP736 were up-regulated upon *V*. *dahliae* infection through RNA-Sequencing. We silenced these two candidate genes in resistant variety Zhongzhimian KV3, respectively (Fig 4C). Severe symptoms were observed in CYP71D- or CYP736-silenced plants compared with vector control plants in response to *V*. *dahliae* (Fig 4A). The DI of silenced plants was increased compared with the control plants (Fig 4B). Shikimate/quinate hydroxycinnamoyl transferase (HCT) functions in the early steps of lignin biosynthesis, and down-regulated HCT in the plants leads to considerable reductions in lignin content [34]. Silenced HCT in the resistant variety Zhongzhimian KV3 resulted in increased susceptibility of resistant cotton to *V*. *dahliae* infection (Fig 4A and 4B). The results showed that these three genes were tightly associated with defense response, suggesting that phenylpropanoid synthesis pathway played an important role in cotton resistance to Verticillium wilt.

### Over-expression of GhSKIP35 contributes to *Arabidopsis thaliana* resistance to Verticillium wilt

**Fig 5:**
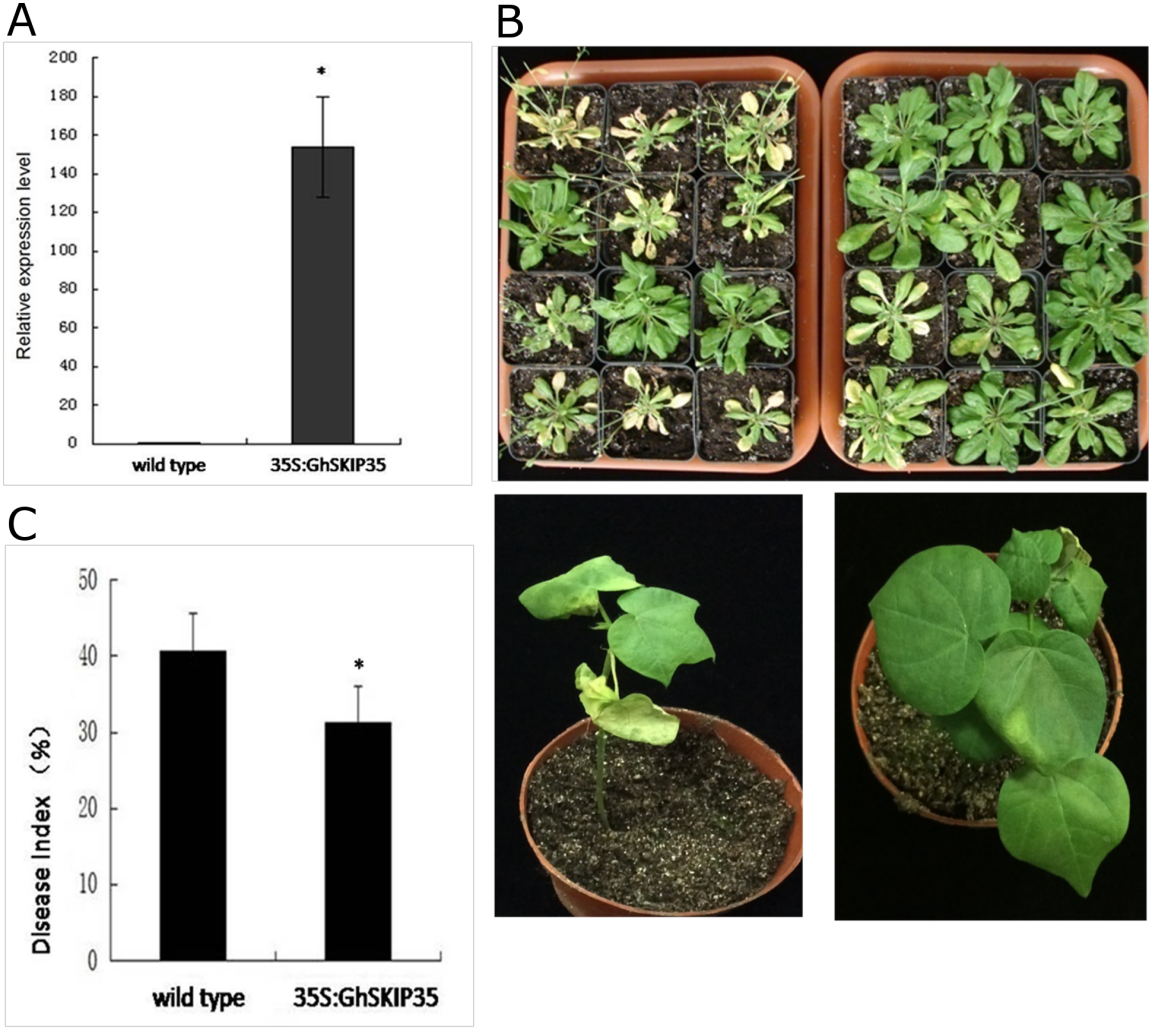
Enhanced disease tolerance of the *Arabidopsis* plants over-expressing GhSKIP35. A, Relative expression level of candidate gene in the transgenic *Arabidopsis* plants leaf tissues was determined through qRT-PCR. The relative expression level of GhSKIP35 was normalized against Ct values for *Arabidopsis* actin gene, which was calculated by the 2^−ΔΔCt^ method (Livak & Schmittgen, 2001). B1, Symptoms of wild-type (left) and GhSKIP35 transgenic plants (right) inoculated with *V*. *dahliae* for 15 days. B2, Negative control-silenced Zhongzhimian KV3 (CLCrV-00) plant (left); GhSKIP35-silenced Zhongzhimian KV3 plant (right) C, The disease index (DI) of wild-type and transgenic *Arabidopsis* plants. The results are presented as mean ±standard deviation (SD) from three replicates with 12 plants per replicate. Asterisk represents the data point that was statistically different from wild-type *Arabidopsis* (p<0.05) analyzed by one-way ANOVA, using the SAS 8.1. The vertical bars indicate standard Deviation.

It has been reported that ubiquitination plays an important role in leucine-rich repeat (NLR) resistance (R) protein-mediated immunity [35, 36]. In cotton, the ubiqutin-protein ligase family is associated with the defense against Verticillium wilt [7]. The SKP1 protein is an evolutionarily conserved subunit of SCF-type E3 ubiquitin ligases that mediate the ubiquitylation of proteins. DEG of SKP1-interacting partner 35 was identified from RNA-sequencing. We amplified the full-length candidate SKP1-interacting partner 35 using RACE-PCR, which was named as GhSKIP35 and contained an open reading frame of 1,863 nucleotides putatively encoding a peptide of 620 amino acid residues [37]. Knockdown of GhSKIP35 resulted in increased susceptibility of resistant cotton to *V*. *dahliae* infection (Fig 5B2). An over-expression strategy was used to assess the function of GhSKIP35. *Arabidopsis* plant was used in the experiment due to technical difficulties and the long duration of cotton transformation. More than 20 transgenic lines constitutively over-expressing GhSKIP35 were obtained. Wild-type and transgenic plants were subjected to *V*. *dahliae* infection using root dipping method. Disease symptoms were examined at 15 dpi. Obvious stunting and more necrotic symptoms were found in wild-type plants compared with transgenic plants (Fig 5B1), showing that the spread of necrosis and stunting in transgenic plants was inhibited by GhSKIP35 over-expression. The DI of the transgenic plants was lower than that of the wild-type plants (Fig 5C). These results suggested that over-expression of GhSKIP35 conferred *Arabidopsis* plants increased tolerance to Verticillium wilt.

## Discussion

Once facing with pathogen attack, plants instigate sophisticated mechanisms in response to pathogen invasion through both constitutive and inducible defenses [38]. In recent years, transcriptome studies have been conducted to reveal the complexity of *V*. *dahliae* infection in cotton [1, 9, 10]. However, the molecular interaction between cotton and *V*. *dahliae* remains largely elusive, especially for upland cotton. Currently, the complete gene profiling of cotton species (*G*. *raimondii*, *G*. *arboretum* and *G*. *hirsutum*) is available [39, 40, 41], and genome database provides convenient for understanding of the cotton defense response to *V*. *dahliae*. RNA-Sequencing technology provides huge amounts of first-hand data, which might involve in cotton defense mechanism against *V*. *dahliae* infection. Therefore, it is urgent to identify and characterize the functions of these first-hand defense responsive genes on a genome-wide scale, which not only helps understand the resistance mechanism, but also provides support for candidate gene selection to breed disease-resistant varieties by genetic engineering. Because of the difficulty of traditional cotton genetic transformation technique, it is a daunting task to identify the functional roles of DEGs during pathogen attack. With the development of VIGS technology, large-scale functional analysis of individual genes by knocking down the expression of endogenous genes becomes realized.

In the present study, we dissected the transcriptional response of *G*. *hirsutum* against *V*. *dahliae* infection using the Illumina-based paired-end deep sequencing platform for genome-scale analyses. Investigations on the relationships between cotton and *V*. *dahliae* revealed that the induction of most defense response occurred early during the interaction [42, 43]. Xu et al [1] selected four time points of *V*. *dahliae* inoculation (such as 4, 12, 24 and 48 h) to analyze the defense response by RNA-sequcing, and the results showed that cotton begins to reponse the pathogen infection after 4 hpi. Here, mock-inoculated and 24 h after *V*. *dahliae* inoculation were taken for analysis and 52 millions of clean reads were generated from each sample. The data were sufficient for quantitative analysis of gene expression. A total of 4,794 DEGs were either up- or down-regulated by *V*. *dahliae* attack. With VIGS technology, we performed functional analysis of several candidate DEGs by suppressing the expressions of endogenous genes in *G*. *hirsutum*. The loss-of-function assays provided important evidence for functional genomic studies of cotton genes.

DEGs involved in “plant-pathogen interaction” and ‘plant hormone signal’ pathways were significantly enriched in the root tissues of resistant *G*. *hirsutum* upon *V*. *dahliae* infection. Rapid changes in the cytosolic concentration of the secondary messenger Ca^2+^ are crucial for downstream responses, such as generation of reactive oxygen species (ROS), activation of calcium-dependent kinase and mitogen-activated protein kinases (MAPKs), and production of defense compounds [27]. A large amount of related DEGs were identified by RNA-sequencing, such as CMLs, CBLs, CaM and EF-hand CBL, which belong to Ca^2+^ signals. MAPKK6 and MAPKKK3 belong to MAPK cascades. Respiratory burst oxidase and ferric-chelate reductase were identified and involved in the regulation of ROS status. The SA and jasmonic acid (JA)-ethylene (ET) hormone pathways play a crucial role in the regulation of defence-gene expression [44]. Recent studies also indicated that complex crosstalk among different classes of hormones might modulate the disease resistance, with outcomes dependent on the pathogen lifestyles and the genetic constitution of the host [45]. Eight DEGs genes functions indicated in SA (*GhPUB*17, *GhTGA*7 and *GhPR*1), JA (*GhJAZ*10 and *GhbHLH*18), ET (*GhEBF*1), cytokinine (*GhE13L*13) and BR (*GhBZR*1) signal pathways were silenced in upland cotton, and demonstrated that these phytohormones pathway related genes are important components in response to *V*. *dahliae* infection [20].

In this study, we identified some important components that are involved in defense response against *V*. *dahliae* infection. WRKY gene family is one group, which might play an important role against Verticillium wilt in cotton. Silencing of GhWRKY2 or GhWRKY29 in Zhongzhimian KV3 resulted in a more severe symptom in the silenced plants compared with the vector control plants, indicating that GhWRKY2 and GhWRKY29 largely contributed to cotton resistance to *V*. *dahliae* infection. Silencing of GhWRKY13 in susceptible variety 86–1 significantly reduced symptoms compared with the vector control plants, revealing that GhWRKY13 served as a negative regulator of resistance to *V*. *dahliae* in upland cotton. As a large gene family, WRKY has received increasing attention due to its roles in plant defense. For example, WRKY29 has been shown to be induced by MAPK cascade in *Arabidopsis* and tobacco, conferring resistance to both bacterial and fungal pathogens [46, 47]. WRKY2 isolated from pepper plays a crucial role in early defense responses to biotic and abiotic stresses [48]. Moreover, the lignin pathway and xylem development can be affected by the grapevine transcription factor WRKY2 in tobacco [49]. In cotton, we also identified two WRKY genes (GhWRKY22, GhWRKY33) from full-length cDNA library, and confirmed that GhWRKY22 and GhWRKY33 were associated with defense response against Verticillium infection [8]. Other WRKY genes, such as GhWRKY11, GhWRKY15, GhWRKY39, GhWRKY40 and GhWRKY44 have been identified to play a significant role in regulating plant pathogen defense responses [50, 51, 52, 53, 54].

The other group was RLKs genes that were important components involved in “plant-pathogen interaction” pathway. Typically, RLKs directly perceive PAMPs by pattern recognition receptors (PRRs) [55], including well characterized LRR-RLKs [56]. *Ve1* was important and suggested as being required for resistance against race 1 of *V*. *dahliae* [57]. The tomato-homologous *Gbve1* gene was up-regulated upon *V*. *dahliae* infection. The expression level of *Gbve1* was increased 3.27-fold in the inoculated resistant cotton cultivar than that in non-inoculated control. It has been found to be involved in defense response against Verticillium [58]. Another important RLKs gene was G-type lectin receptor kinase, *GhGsSRK*. We silenced GhGsSRK in resistant cotton plants, and the result showed that it played an important role against Verticillium wilt via VIGS analysis. Chen et al. [59] identified a G-type lectin receptor kinase (Pi-d2) from rice, which facilitates resistance against the fungal pathogen *Magnaporthe grisea*, the causal agent of rice blast.

Other important component was cytochrome P450 (CYPs) gene family. It has been reported that hundreds of CYPs are involved in the phenypropanoid metabolism [60]. Phenylpropanoid pathway was confirmed to play an important role in response to *V*. *dahliae* [1, 61, 62]. CYP71D and CYP736 were up-regulated upon *V*. *dahliae* attack. They are involved in ferulate 5-hydroxylase (F5H) synthesis. It has been reported that F5H is cytochrome P450-dependent monooxygenase in phenylpropanoid metabolisms. Over-expression of F5H enhances lignin syringyl contents, which may function in response to various stresses [63, 64]. Knockdown of CYP71D and CYP736 in resistant upland cotton variety Zhongzhimian KV3, resulting in the loss of resistance to Verticillium wilt, that might lead to affect F5H synthesis.

In conclusion, our data provided significant insights into the molecular mechanism of cotton responses to *V*. *dahliae*, especially a better understanding of early defense response of upland cotton to *V*. *dahliae*. We screened several important pathways and components involved in upland cotton defense responses against *V*. *dahliae* infection by using VIGS and over-expression stratigies for gene functional characterization. This study provided an important strategy of high-throughput screening and identification of genes involved in defense responses, and our data also offered candidate genes for further genetic engineering of Verticillium wilt tolerance in upland cotton.

## Supporting information

S1 Table Primers used for qRT-PCR. (DOCX)

S2 Table Primers used for pCLCrV-cloning. (DOCX)

S3 Table Primers used for qRT-PCR of silenced plants or over expression plants. (DOCX)

S4 Table Summary of the read information from Zhongzhimian KV3 at 24 h post inoculation (hpi) and non-inoculated samples. (DOCX)
