# Supplementary material for: Large-scale identification of Gossypium hirsutum genes associated with Verticillium dahliae by comparative transcriptomic and reverse genetics analysis
Source: PLoS One. 2017 Aug 2;12(8):e0181609. doi: 10.1371/journal.pone.0181609 (PMC5540499; doi:10.1371/journal.pone.0181609)
Supplement: S1 Table — (DOCX) [file pone.0181609.s001.docx]

**S1Table. Primers used for qRT-PCR**

| **Name** | **Forward primer sequence (5’-3’)** | **Reverse primer sequence(5’-3’)** |
| --- | --- | --- |
| *Ubiquitin* | AAGACGAAGAACAAGGTGAA | GCTCGGATACGATTGATAAC |
| *GhFLS2* | GAGTTATCTCGGAAAGTTCAAAGC | GATCGGAGATGAAGTGACCCA |
| *GhVe* | CTTCCACAATACGCCACC | AGAAGAAATAGGCAAACTGG |
| *GhMRH1* | GGCCAATCTGAAGATGCAATG | TCAGCGGAGACAGGAGTAGGAG |
| *GhWRKY29* | TTCAGATACAACGCAAGATGGTC | GGCTCCAATGTAAGTGGTTCG |
| *GhWRKY16* | GGGTACGAGTTGCGTTTAGAAT | TACCTTGGATTGGGGCTGTTC |
| *GhLRRC* | AATCGTTGACGGCAAGGTTAC | TGAGCTTGTTGAAGTTGGCATT |
| *GhSLSG* | TCTTCAGGGGTAATCAACACGA | CCGGCCATAAATGATTTCTAAC |
| *GhBZR1* | TGCCTCCACTCAGAATCTCAAA | TGGGGCAGAAACAGCATAAA |
| *GhJAZ* | TTTTACTGCGGACAAGTGATTG | AGTAGGAACCTGATCGTTGGAAT |
| *GhSAPK1* | CAATGCAAATATGCCACCG | GAACCTCAGGAGCAATGTAAGC |
| *GhPP2C* | CATCTTTTCCCATTTCTCCACA | CCGAGATTCGCCACATACAA |
| *GhEBF* | TCCCTGATGAATGCCTTGTTG | TCCTTGACCACCTTTGATGACT |
| *GhCYP71D10* | TCAACGAGGAAGGAGGTGCTA | CCCCAAAACCATTCTCAAAGTC |
| *GhDBTNBT* | TATGGCAGAGTGGAGTTACCGA | GGTTTGTGATTTGTGGTCCGT |
| *GhSLP* | ACCGCAGCAACAGATCAAGTG | TTCTTCGGTGGCTGTAGGGTC |
| *GhRCHY1* | AGAACTGGAGGCGAGGAGAAC | AGCAAATAGGGCAATCGTGGT |
| *GhCUL1* | GAAATCAAAGATGCTGTATTGTCCC | TTTCATAGAGCCTTGCCCAAC |
| *GhRC4* | ACCCTACAAATGTTCAGCTCCC | TTTCTCCATACGACCATCACG |
