# Supplementary material for: Large-scale identification of Gossypium hirsutum genes associated with Verticillium dahliae by comparative transcriptomic and reverse genetics analysis
Source: PLoS One. 2017 Aug 2;12(8):e0181609. doi: 10.1371/journal.pone.0181609 (PMC5540499; doi:10.1371/journal.pone.0181609)
Supplement: S2 Table — (DOCX) [file pone.0181609.s002.docx]

**S2 Table. Primers used for pCLCrV-cloning**

| **Name** | **Forward primer sequence (5’-3’)** | **Reverse primer sequence(5’-3’)** |
| --- | --- | --- |
| *GhFLS2* | GGACTAGTTAATCGTCTGTCAGGGCA | AAGGCGCGCCGCTGGCTTCCAAGTGTCT |
| *GhGsSRK* | GGACTAGTGGAAAAGGAGAAACCAATC | AAGGCGCGCCCTGTCAAGCGAACCATTT |
| *GhWRKY2* | GGACTAGTCTTTGGGTCACTGCTACA | AAGGCGCGCCAGGCTTGGCACTACACTT |
| *GhWRKY29* | GGACTAGTGAGAAAGAGGGCAGAACA | AAGGCGCGCCCAAGTCCGTAACATCGTC |
| *GhWRKY13* | GGACTAGTCTGGGAACCAAGAAAAGT | AAGGCGCGCCTCCTGCGACGGAGAATGA |
| *GhCYP71D* | GGACTAGTAAAAGGGGCATAGCATTC | AAGGCGCGCCTCACCGAGTTTCGTTGTA |
| *GhCYP736* | GGACTAGTATAGAACAGGGGAAGTCA | AAGGCGCGCCCACCAAAAGCCTCAGTCA |
| *GhHCT* | CCTTAATTAAATCCTAAAGCCATCCGTT | AAGGCGCGCCGTGATTTGTGGTCCGTGA |
| *GhSKIP35* | GGACTAGTATCTTGCCTTGGGTTTTG | AAGGCGCGCCTGCCTCTGACCAGTGTTC |

Underlined are restriction sites: *Spe* I: ACTAGT; *Asc* I: GGCGCGCC; *Pac* I: TTAATTAA
