# Supplementary material for: Large-scale identification of Gossypium hirsutum genes associated with Verticillium dahliae by comparative transcriptomic and reverse genetics analysis
Source: PLoS One. 2017 Aug 2;12(8):e0181609. doi: 10.1371/journal.pone.0181609 (PMC5540499; doi:10.1371/journal.pone.0181609)
Supplement: S3 Table — (DOCX) [file pone.0181609.s003.docx]

**S3 Table Primers used for qRT-PCR of silenced plants or over expression plants**

| **Name** | **Forward primer sequence (5’-3’)** | **Reverse primer sequence(5’-3’)** |
| --- | --- | --- |
| *Ubiquitin* | AAGACGAAGAACAAGGTGAA | GCTCGGATACGATTGATAAC |
| *Actin* | AGTGGTCGTACAACCGGTATTGT | GATGGGCATGAGGAAGAGAGAAAC |
| *GhFLS2* | GAGTTATCTCGGAAAGTTCAAAGC | GATCGGAGATGAAGTGACCCA |
| *GhWRKY2* | TGTTCGCACAAATCCTTCTG | AGGCTTGGCACTACACTTGG |
| *GhWRKY29* | TTCAGATACAACGCAAGATGGTC | GGCTCCAATGTAAGTGGTTCG |
| *GhWRKY13* | TGGGAACCAAGAAAAGTAGTGG | CGCCCTTCGTAGGTGGTAAT |
| *GhCYP71D* | TCAACGAGGAAGGAGGTGCTA | CCCCAAAACCATTCTCAAAGTC |
| *GhCYP736* | TAGAACAGGGGAAGTCAACGAA | CTTGGAATTAGCAAAGGTAGAGGA |
| *GhHTC* | TATGGCAGAGTGGAGTTACCGA | GGTTTGTGATTTGTGGTCCGT |
| *GhSKIP35* | GAGTGTCTAGTGGAAGAGGGTAATG | CTGGAAGTAGCGGCAGTGAG |
