# Supplementary material for: Large-scale identification of Gossypium hirsutum genes associated with Verticillium dahliae by comparative transcriptomic and reverse genetics analysis
Source: PLoS One. 2017 Aug 2;12(8):e0181609. doi: 10.1371/journal.pone.0181609 (PMC5540499; doi:10.1371/journal.pone.0181609)
Supplement: S4 Table — (DOCX) [file pone.0181609.s004.docx]

**S4 Table. Summary of the read information from Zhongzhimian KV3 at 24 h post inoculation (hpi) and mock-inoculated samples**

| Sample | Total reads | Total maped reads | unique match | multi-position match |
| --- | --- | --- | --- | --- |
| KV3 (mock-inoculated) -1 | 53189643 | 34872170 | 31708233 | 3163937 |
| KV3 (mock-inoculated) -2 | 53008273 | 34976042 | 31715901 | 3260141 |
| KV3 (inoculated, 24 h)-1 | 52358923 | 34836807 | 32906855 | 1929951 |
| KV3 (inoculated, 24 h)-2 | 52593617 | 34498435 | 32797541 | 1700894 |
